# A Narrative Review of Preventive Central Lymph Node Dissection in Patients With Papillary Thyroid Cancer - A Necessity or an Excess

**DOI:** 10.3389/fonc.2022.906695

**Published:** 2022-06-29

**Authors:** David D. Dolidze, Alexey V. Shabunin, Robert B. Mumladze, Arshak V. Vardanyan, Serghei D. Covantsev, Alexander M. Shulutko, Vasiliy I. Semikov, Khalid M. Isaev, Airazat M. Kazaryan

**Affiliations:** ^1^Department of Surgery, Russian Medical Academy of Continuous Professional Education, Moscow, Russia; ^2^Department of Surgery, S.P. Botkin City Clinical Hospital, Moscow, Russia; ^3^Department of Faculty Surgery №2, I.M.Sechenov First Moscow State Medical University, Moscow, Russia; ^4^Department of Gastrointestinal Surgery, Østfold Hospital Trust, Grålum, Norway; ^5^Department of Surgery, Fonna Hospital Trust, Odda, Norway; ^6^Intervention Centre, Oslo University Hospital – Rikshospitalet, Oslo, Norway; ^7^Department of Surgery №1, Yerevan State Medical University after M.Heratsi, Yerevan, Armenia

**Keywords:** papillary thyroid cancer, preventive central lymph node dissection, hypocalcemia, recurrent laryngeal nerve paresis, metastasis, cancer recurrence

## Abstract

**Objective:**

This review article summarises the latest evidence for preventive central lymph node dissection in patients with papillary thyroid cancer taking into account the possible complications and risk of recurrence.

**Background:**

Papillary thyroid cancer is the most frequent histological variant of malignant neoplasms of the thyroid gland. It accounts for about 80-85% of all cases of thyroid cancer. Despite good postoperative results and an excellent survival rate in comparison with many other malignant diseases, tumor metastases to the cervical lymph nodes are frequent. Most researchers agree that the presence of obvious metastases in the lymph nodes requires careful lymph node dissection. It was suggested to perform preventive routine lymphadenectomy in all patients with malignant thyroid diseases referred to surgery.

**Methods:**

It was performed the literature review using the “papillary thyroid cancer”, “central lymph node dissection”, “hypocalcemia”, “recurrent laryngeal nerve paresis”, “metastasis”, “cancer recurrence” along with the MESH terms. The reference list of the articles was carefully reviewed as a potential source of information. The search was based on Medline, Scopus, Google Scholar, eLibrary engines. Selected publications were analyzed and their synthesis was used to write the review and analyse the role of preventive central lymph node dissection in patients with papillary thyroid cancer.

**Conclusions:**

The necessity of preventive central lymph node dissection in patients with differentiated papillary thyroid carcinoma is still controversial. There is much evidence that it increases the frequency of transient hypocalcemia. Due to the fact that this complication is temporary, its significance in clinical practice is debatable. It can also be assumed that an extant of surgery in the neck area is associated with an increased risk of recurrent laryngeal nerve injury. However, most studies indicate that this injury is associated more with thyroidectomy itself than with lymph node dissection. Recurrent laryngeal nerve dysfunction is also a temporary complication in the vast majority of cases. At the same time, a large amount of data shows that central lymph node dissection reduces the risk of thyroid cancer recurrence in two times.

## Introduction

Papillary thyroid cancer is the most frequent histological variant of malignant neoplasms of the thyroid gland. It accounts for about 80-85% of all cases of thyroid cancer. At the same time, the 10-year survival rate is more than 90% (1–4). Despite good postoperative results and an excellent survival rate in comparison with many other malignant diseases, tumor metastases to the cervical lymph nodes occur on average in 33% of patients (5). The most common area of ​​metastases for papillary thyroid cancer is group VI (central) of the cervical lymph nodes (6). Given the high incidence of metastases to the cervical lymph nodes, prophylactic central lymph node dissection may be a logical procedure to reduce the risk of postoperative tumor recurrence (7). Nevertheless, the accumulated experience in the treatment and research of thyroid cancer has led to the question of the rationality of preventive central lymphadenectomy as a routine procedure (8). This is primarily due to the fact that lymph node dissection increases the risk of possible complications such as hypoparathyroidism and recurrent laryngeal nerve injury (9). Most researchers agree that the presence of obvious metastases in the lymph nodes requires careful lymph node dissection. The necessity of preventive or routine lymphadenectomy for all patients with malignant thyroid diseases remains controversial. The current review provides an analysis of the accumulated knowledge of preventive lymphadenectomy as well as its benefits and disadvantages.

## Methods

**Figure 1:**
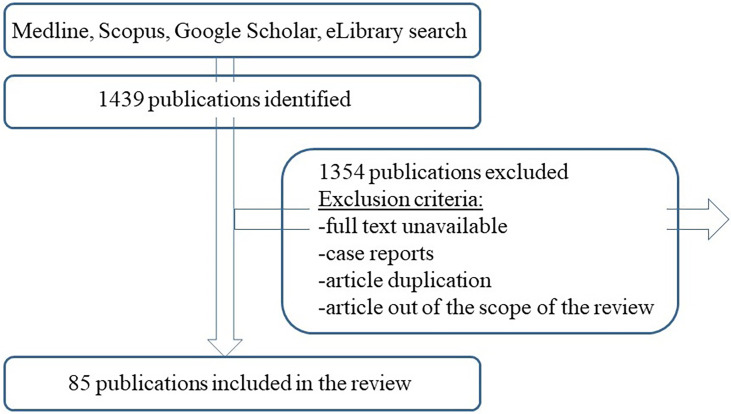
Flow chart of the review.

DDD, SDC, KMI performed the literature search using the “papillary thyroid cancer”, “central lymph node dissection”, “hypocalcemia”, “recurrent laryngeal nerve paresis”, “metastasis”, “cancer recurrence” along with the MESH terms in English and Russian languages with no year limitation. The reference list of the articles was carefully reviewed as a potential source of information. The search was based on Medline, Scopus, Google Scholar, eLibrary engines. Selected publications were analyzed and their synthesis was used to write the review and analyse the role of preventive central lymph node dissection in patients with papillary thyroid cancer (**Figure 1**).

## Central Lymph Node Dissection and its Advantages

**Figure 2:**
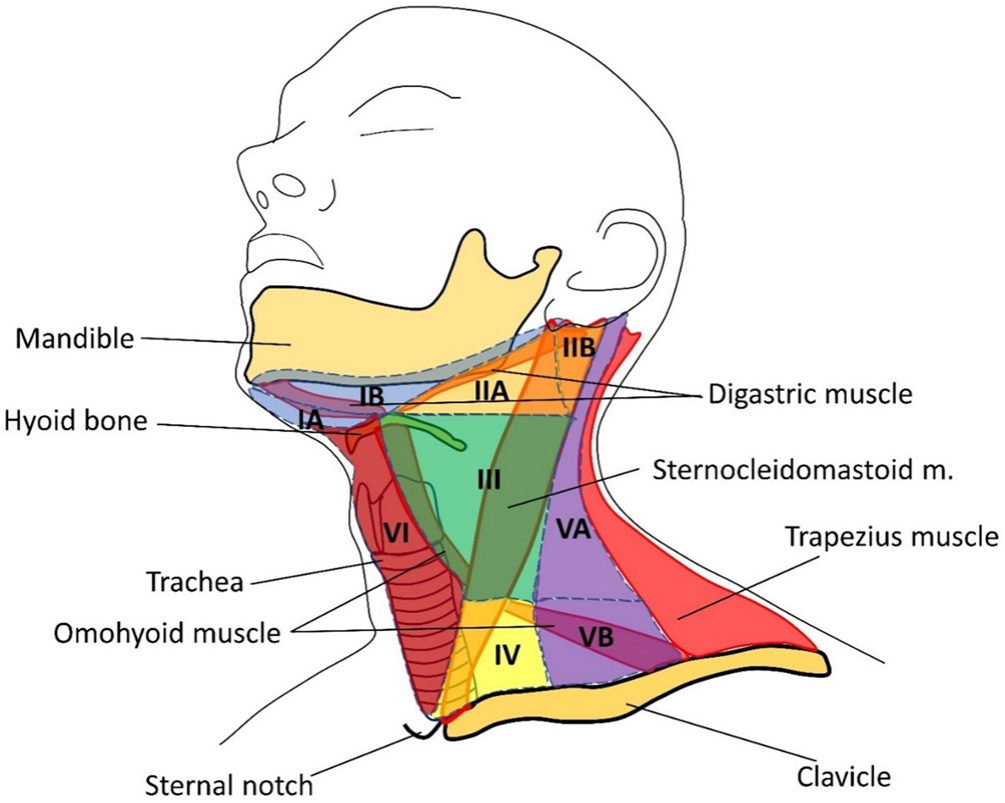
Groups of cervical lymph nodes. I – below the body of the lower jaw; IA: group of chin nodes; IB: group of submandibular lymph nodes; II: upper jugular; IIA: upper jugular anterior; IIB: upper jugular posterior; III: middle jugular; IV: lower jugular; V: posterior (lateral) triangle of the neck; VA: accessory; VB: supraclavicular; VI: anterior space of the neck.

Central lymph node dissection involves the removal of group VI lymph nodes. The dissection is performed from the hyoid bone to the suprasternal notch along the carotid arteries, trachea, and prevertebral fascia (**Figure 2**, **Table 1**). Most often metastases are found in this anatomical area on histological examination, at the same time, they are not always visible on ultrasound, CT, or even during surgery (10–13).

**Table 1:** Groups and localization of lymph nodes.

Group	Subgroup	Localization	Likelihood of metastasis
I: below the body of the lower jaw	IA	group of chin nodes	5-9%
	IB	group of submandibular lymph nodes	
II: upper jugular - from the level of the base of the skull to the level of the lower edge of the hyoid bone	IIA upper jugular anterior	anteriorly from the posterior edge of the internal jugular vein	47-60%
	IIB superior jugular posterior	posteriorly from the posterior edge of the internal jugular vein	8-27%
III: middle jugular	–	from the level of the lower edge of the hyoid bone to the level of the lower edge of the cricoid cartilage of the larynx	67-74%
IV: lower jugular	–	from the level of the lower edge of the cricoid cartilage of the larynx to the clavicles	61-71%
V: the posterior (lateral) triangle of the neck	VA	In front is delimited by the posterior edge of the sternoclavicular nipple muscle, behind-by the anterior edge of the trapezius muscle, from below - by the clavicle. Inferior border of cricoid separates VA and VB	3-20%
	VB		8-48%
VI (central) anterior space of the neck	–	pretracheal and paratracheal lymph nodes pre-laryngeal lymph nodes	40-60%

The main advantages of preventive central dissection are: removal of subclinical metastases, improvement of postoperative survival by reducing the frequency of cancer recurrences, reduction in the number of repeated operations for thyroid cancer, significantly more noticeable decrease in thyroglobulin in the postoperative period (2, 14). A meta-analysis of 14 studies involving 4573 patients showed that, on average, lymph node group VI metastases occur in 33% of cases (5). An important factor is also the frequency of metastases outside the central group. A systematic review and meta-analysis of 23 studies, which included 18741 patients, showed that, on average, metastases to the lateral group of lymph nodes occur in 20.9% of patients (15). However, in other studies, the frequency of metastases reaches 40-90% (14, 16, 17). Lymph node metastases are responsible for 75% of locoregional relapses and 50% of deaths (18).

An important reason for performing preventive central lymphadenectomy is the lack of reliable methods for detecting metastases in lymph nodes before operation. The sensitivity of preoperative neck ultrasound to detect lymph nodes is only 46-88%, depending on the size of lymph nodes and technical capabilities of the ultrasound equipment (19–21). CT of the neck also has low rate of sensitivity (74% to 82%) (15). The sensitivity of this method is noticeably reduced in cases when the lymph nodes are less than 9 mm in diameter, which happens in the vast majority of cases (22). According to the results of a meta-analysis of 9 studies involving 1691 patients, the combination of ultrasound examination and CT in detecting metastases to lymph nodes gives a sensitivity of 69% (23).

Another important aspect is the lack of standards for assessing lymph nodes by ultrasound and CT (15, 24). In fact, an increased lymph nodes do not always indicate on their metastatic involvement. In 20% of cases, an increased lymph nodes may be detected against the background of an inflammatory process, mainly Hashimoto’s thyroiditis (25, 26). A meta-analysis of 71 studies, which included 44034 patients, showed that thyroid cancer with or without lymph node involvement is much less common in patients with Hashimoto’s thyroiditis. Hashimoto’s thyroiditis also reduces the risk of cancer recurrence in patients operated on for malignant tumors of the thyroid gland (27, 28).

Staging of thyroid cancer depends on the evaluation of lymph nodes. Many prognostic scales are also accounted for by the involvement of lymph nodes as one of the main criteria of disease severity and negative prognosis (29–31).

## Disadvantages of Central Lymph Node Dissection

The main argument in favor of refusing preventive lymphadenectomy is the higher frequency of postoperative complications. The main complications after thyroidectomy with central lymph node dissection are transient and permanent hypoparathyroidism, transient and permanent recurrent laryngeal nerve paresis. According to a meta-analysis of 14 articles that included 4573 patients, the incidence of permanent hypoparathyroidism, recurrent laryngeal nerve paresis and cancer recurrence is 1.1%, 0.5% and 2.8%, respectively (5). The frequency of postoperative hypoparathyroidism is 36.1-42.4%, but it decreases to 1.1-3.9% after 6 months -1 year (32, 33). Risk factors for postoperative hypocalcemia are female, bilateral lymphadenectomy, high ligation of the thyroid arteries, large thyroid gland, low levels of preoperative calcium and parathyroid hormone, autotransplantation of the parathyroid gland (32–35). At the same time, if the parathyroid gland was removed during surgery, its reimplantation with adequate blood supply significantly reduces the risk of hypocalcemia (34, 36). Due to the fact that permanent hypocalcemia is a rare complication, it is difficult to reliably assess the effect of lymphodissection because a small number of cases creates difficulties for adequate statistical analysis. To assess the relationship between lymphodissection and permanent hypocalcemia, an analysis of a large database at the national level is required. The heterogeneity of the data also makes it difficult to perform a meta-analysis that would reliably indicate the effect of central lymph node dissection on the frequency of hypocalcemia (37). The hypocalcemia in the postoperative period has many reasons. A meta-analysis of 23 studies with a total of 877356 patients indicates 12 risk factors for the development of postoperative hypocalcemia, such as hypoparathyroidism, thyroidectomy, hypomagnesemia, vitamin D deficiency, female, thyroid cancer, thyroiditis, retrosternal goiter, parathyroid attachment, central lymph node dissection, lateral lymph node dissection, dissection of surrounding thyroid gland tissues (38).

Seo and co-authors compared 52707 thyroidectomies and 139626 thyroidectomies with central lymph node dissection and found that the frequency of permanent hypocalcemia was higher when performing central lymph node dissection (5.4% compared to 4.6%) (39). Another important finding was the frequency of hypocalcemia depending on the operational activity of the hospital. In hospitals where less than 200 surgical interventions are performed per year, the frequency of hypocalcemia was 6.0-6.5%, in hospitals where 200-799 thyroidectomies are performed per year the frequency of hypocalcemia varied from 3.2% to 7.4%, and in hospitals where more than 800 operations are performed per year – only 3.3% (39). Thus, it is reliably known that the volume of surgical intervention and an experience of surgeons are interrelated with the frequency of postoperative hypocalcemia.

Transient and permanent disorders of recurrent laryngeal nerve function after thyroidectomy remain a serious problem. Postoperative laryngeal paresis occurs in 3.28-27.8% of cases (40–42). In most cases, recurrent laryngeal nerve paresis is transient, because 94.6% of patients have a complete restoration of the voice (43). Lyomasa and co-authors noted that the voice impairment due to injury of the upper laryngeal nerve decreases to zero in the 6th month after surgery in comparison with the first postoperative day. As far as recurrent laryngeal nerve, the authors noted that paresis occurs in 27.8% of patients on the first postoperative day, but decreases to 6.6% after 6 months (41). An important observation is that atypical variants of recurrent laryngeal nerve occur in 24.4% and require a careful identification during surgery (44, 45). In general, the risk of nerve damage is low and more common during repeated operations due to fibrosis of surrounding tissues, which makes it difficult to detect the nerve (46). The question whether central lymph node dissection is a risk factor for transient paresis and laryngeal paralysis remains controvercial because there are radically different data (46–48). Machens and co-authors, analyzing the data of 102 pediatric patients who underwent thyroidectomy with central lymph node dissection, noted an increased rate of only transient laryngeal paresis (6).

Another important problem is cancer metastasis to the lymph nodes along the recurrent laryngeal nerve, which occurs in 8.65% of cases and requires meticulous dissection in that area (49). Manipulations in the nerve area are naturally associated with the risk of its dysfunction in the postoperative period, even if the nerve was not directly injured during the operation. In some patients, thyroid cancer nerve involvement is also noted, which is associated with neuropaxy (50).

Several systematic reviews compared incidence of complications in two groups of patients: those who underwent thyroidectomy and thyroidectomy in combination with central lymph node dissection (**Table 2**). The majority of authors agree that the risk of transient hypocalcemia increases by 1.5-2.5 times, while the risk of paresis does not differ between the two groups (**Table 2**).

**Table 2:** Systematic meta-analyses reviews comparing risk hypocalcemia and recurrent laryngeal nerve paresis after thyroidectomy and thyroidectomy with preventive central lymph node dissection.

Author, year, reference	Number of studies, patients	Hypocalcemia	Recurrent laryngeal nerve paresis
Chisholm (2009) (51)	5 studies, 1132 patients	For every 7.7 thyroidectomies with central lymph node dissection there was an additional case of transient hypocalcemia compared with only thyroidectomy. The risk of permanent hypocalcemia was not increased	The risk of paresis was not higher
Shan (2012) (52)	16 studies, 3558 patients	After thyroidectomy with central lymph node dissection transient hypocalcemia was diagnosed more often (31%) than after thyroidectomy alone (16%). The frequency of permanent hypocalcemia did not differ	The rate of recurrent laryngeal nerve paresis was higher after thyroidectomy with central lymph node dissection (5.2% compared to 2.9% after thyroidectomy), but the difference was not statistically significant
Lang (2013) (53)	14 studies, 3331 patients	After thyroidectomy with central lymph node dissection transient hypocalcemia was diagnosed more often than after thyroidectomy alone (26.0% versus 10.8%)	The risk of paresis was not higher
Wang (2013) (54)	6 studies, 1342 patients	After thyroidectomy with central lymph node dissection transient hypocalcemia was diagnosed more often than after thyroidectomy alone	The risk of paresis was not higher
Zhu (2013) (55)	9 studies, 2298 patients	Thyroidectomy with centarl lymphodissection was associated with transient hypocalcemia	The risk of paresis was not higher
Liang (2017) (56)	23 studies, 6823 patients	The risk of transient and permanent hypocalcemia was higher after thyroidectomy with central lymphodissection (p<0.01).	The risk of transient paresis of the laryngeal nerve was higher after thyroidectomy with central lymph node dissection (p = 0.023)
Sison (2019) (57)	8 research 13428 patients	Transient hypocalcemia was more common in thyroidectomy with lymph node dissection (5.72% vs. 3.34%)	The risk of permanent laryngeal nerve paresis was not higher
Su (2019) (58)	4 studies, 727 patients	There was no difference between the two groups	The risk of paresis was not higher

It is worth noting that the risk of complications is significantly reduced in hospitals with a large number of surgical interventions per year. Thus, it is rational to perform thyroidectomy with central lymph node dissection in large institutions where a specialist performs at least 50 operations per year (59–62).

## Recommendations of Professional Communities and the Risk of Cancer Recurrence

National and international communities have different assessments of the need for central lymph node dissection in thyroid cancer. These recommendations are presented in **Table 3**.

**Table 3:** Recommendations for central lymph dissection.

Community/Year	Recommendation
European Society of Endocrine Surgeons	Patients at high risk of cancer recurrence (T3/T4 tumor, elderly age, men, bilateral tumor location, multifocal tumor, enlarged lymph nodes). The operation should be performed in specialized departments (63)
American Thyroid Association (2016)American Association of Endocrine Surgeons Guidelines (2020)	Patients with T3/T4 tumor, involvement of lateral lymph nodes of the neck or the next methods of treatment depends upon lymph node dissection (64) During initial thyroidectomy for PTC, the central compartment should be assessed for suspicious lymphadenopathy. If clinical or imaged LNM is present (ie, macroscopic disease), a therapeutic CND is recommended (13).
Russian recommendations (2018)	Primary tumor T3 or T4, preoperatively verified metastases in the lateral lymph nodes of the neck (CN1B) (65)
National Comprehensive Cancer Network (National Comprehensive Cancer Network) (2016)	Patients with T3/T4 tumors, but the risk of hypoparathyroidism and recurrent laryngeal nerve damage must be taken into account (66)
British Thyroid Association (2014)	The benefits for a high-risk patient are unclear, so decision-making should be individual. Preference should be given to bilateral central lymph dissection rather than unilateral (67)
Korean Society of Thyroid Surgeons (2016)	Primary tumor T3 or T4 (68)
Japanese Society of Thyroid Surgeons/Japan Association of Endocrine Surgeons (2020)	Always performed (69, 70)

The majority of surgeons estimate the size of tumor as T3/T4 for recommendation of lymph node dissection. According to Japanese professional communities central lymph node dissection should always preventively be performed. Japanese institutions have accumulated extensive experience in performing preventive lymph node dissections. Out of 4301 patients in whom lymphogenic metastases were not detected preoperatively, preventive lymph node dissection revealed N1a in 2548 (59%) cases. During follow-up cancer recurrence was diagnosed only in 1.2% of cases. At the same time, the 10-year and 20-year survival rates were 99.1 and 98.2%, respectively (71). In fact, there are two approaches: the “western” way of treatment, where preference is given to thyroidectomy followed by radioactive iodine ablation, and the “eastern” way of treatment, where preference is given to preventive lymph node dissection (72, 73).

There were also attempts to find the “optimal” group of patients for whom preventive lymph node dissection is beneficial. The risk group for detecting metastases in lymph nodes in the absence of information about their presence before surgery includes the following factors: male sex, age less than 45 years, multiple tumors, tumor size more than 2 cm, localization in the center of the lobe or at the lower pole, invasion into vessels, spread outside the thyroid gland capsule (74). Other factors may include isthmus location of the tumor, male sex, age less thatn 45 years, tumor adjacent to dorsal membranes, and irregular borders (75, 76). Most meta-analyses up to 2013 indicated a lack of data that central lymph node dissection somehow reduces the risk of locoregional cancer recurrence (**Table 4**). Further studies have shown that preventive central lymph node dissection reduces the risk of relapse by about half.

**Table 4:** Meta-analyses assessing the frequency of cancer recurrence depending on the method of surgery.

Author, year, link	Number of studies and patients	Risk of recurrence
Zetoune (2010) (77)	5 studies, 1264 patients	Central lymph node dissection did not reduce the risk of tumor recurrence
Shan (2012) (52)	16 studies, 3558 patients	Central lymph node dissection did not reduce the risk of tumor recurrence
Lang (2013) (53)	14 studies, 3331 patients	Central lymph node dissection did not reduce the risk of tumor recurrence
Wang (2013) (54)	6 studies, 1342 patients	The risk of recurrence after lymph node dissection is significantly reduced, 31 lymph node dissection prevents one recurrence
Zhu (2013) (55)	9 studies, 2298 patients	Central lymph node dissection did not reduce the risk of tumor recurrence
Liang (2017) (56)	23 studies, 6823 patients	The Risk of recurrence was lower after the Central lymph node dissection
Sison (2019) (57)	8 research 13428 patients	The Risk of recurrence was lower in the prophylactic lymph node dissection group (1.96% versus 2.60%)
Su (2019) (58)	4 studies, 727 patients	The Risk of recurrence was lower after the Central lymph node dissection
Liu (2019) (78)	25 studies, 7052 patients	The addition of central neck dissection to thyroidectomy resulted in a greater reduction in risk of local recurrence than thyroidectomy alone, especially preventing central neck recurrences. Bilateral central neck dissection in patients with papillary thyroid cancer more than 1 cm was necessary.

## Comparison of Surgery and Radioactive Iodine Treatment

The role of radioactive iodine (RAI) treatment in differentiated thyroid cancer is controversial. It seems that RAI is a relatively safe treatment option in moderate doses (79). Recent studies demonstrate that in patients with low-risk thyroid cancer undergoing thyroidectomy, a follow-up strategy that did not involve the use of radioiodine was noninferior to an ablation strategy with radioiodine regarding the occurrence of functional, structural, and biologic events at 3 years (80). Follow-up at 6-18 months is based on serum thyroglobulin, thyroglobulin-antibody determination and neck ultrasonography as the cancer recurrence rate is approximately 3% (81). It seems that low-risk thyroid cancer can be monitored effectively as it does not cause significant mortality and morbidity (82). The recent advances in our understanding of thyroid cancer has allowed to expand the definition of “low risk” as demonstrated in **Table 5** (64, 82).

**Table 5:** Low risk definition for recurrence and mortality of well differentiated thyroid cancer.

Low-risk for recurrence	Low-risk for mortality
No local or distant metastases	Age cut-off <55 years of age at diagnosis
All macroscopic tumor has been resected	Minor extrathyroidal extension detected only on histological examination has no impact on either T category or overall stage
No tumor invasion of loco-regional tissues or structures	T3a tumors >4 cm confined to the thyroid gland, any N, M0
No aggressive cyto-type (e.g., tall cell, hobnail variant, columnar cell carcinoma)	T3b tumor of any size with gross extrathyroidal extension into strap muscles only (sternohyoid, sternothyroid, thyrohyoid, or omohyoid muscles), any N, M0
If RAI given, there are no RAI-avid metastatic foci outside the thyroid bed on the first post treatment WBS	
No vascular invasion	

Moreover, further research has demonstrated theat several other features can be included as criteria for low-risk: intra-thyroidal encapsulated follicular variant of papillary thyroid cancer, papillary thyroid cancer with: clinical N0 or ≤5 pathologic N1 micro-metastases (<0.2 cm in largest dimension), intra-thyroidal well differentiated follicular thyroid cancer with capsular invasion and no or minimal (<4 foci) vascular invasion, intra-thyroidal papillary microcarcinoma, unifocal or multifocal, including BRAFV600E mutated (if known) (25, 64, 83).

Moreover, the clinical response to RAI therapy for thyroidectomy with central lymph node dissection is not superior to thyroidectomy alone in cN0 papillary thyroid cancer patients (84).

## Conclusions

The necessity of preventive central lymph node dissection in patients with differentiated papillary thyroid carcinoma remains controversial. There is much evidence that it increases the frequency of transient hypocalcemia. Due to the fact that this complication is temporary, its significance in clinical practice is debatable. It can also be assumed that an extant of surgery in the neck area is associated with an increased risk of recurrent laryngeal nerve injury, however, most studies indicate that this injury is associated more with thyroidectomy itself than with lymph node dissection. Recurrent laryngeal nerve dysfunction is also a temporary complication in the vast majority of cases. At the same time, a large amount of data shows that central lymph node dissection reduces the risk of thyroid cancer recurrence by about half. It is also important to emphasize that it is rational to perform such operations in medical centers with a large number of thyroid interventions, which significantly reduces the risk of complications. Further research will help to determine an individual approach in the selection of patients for whom central lymph node dissection is beneficial. Taking into account the absence of reliable criteria for determination of metastatic lymph node lesions before operation, low accuracy of ultrasound examination and CT, the risk of cancer recurrence, central lymph node dissection remains an urgent method of thyroid cancer recurrence prevention.

## Author Contributions

(I) Conception and design: DD. (II) Administrative support: DD, AS, VS, AK. (III) Literature search and systematisation: DD, KI, SC. (IV) Data analysis and interpretation: All authors. (V) Manuscript drafting: DD, AK, SC, VS. (VI) Critical revision and final approval of manuscript: All authors.

## Conflict of Interest

The authors declare that the research was conducted in the absence of any commercial or financial relationships that could be construed as a potential conflict of interest.

## Publisher’s Note

All claims expressed in this article are solely those of the authors and do not necessarily represent those of their affiliated organizations, or those of the publisher, the editors and the reviewers. Any product that may be evaluated in this article, or claim that may be made by its manufacturer, is not guaranteed or endorsed by the publisher.
